# Anastrozole Protects against Human Coronavirus Infection by Ameliorating the Reactive Oxygen Species–Mediated Inflammatory Response

**DOI:** 10.3390/antiox13010116

**Published:** 2024-01-17

**Authors:** Eun-Bin Kwon, Buyun Kim, Young Soo Kim, Jang-Gi Choi

**Affiliations:** Korean Medicine (KM) Application Center, Korea Institute of Oriental Medicine (KIOM), Dong-gu, Daegu 41062, Republic of Korea; wrld2931@kiom.re.kr (E.-B.K.); bykim@kiom.re.kr (B.K.)

**Keywords:** human coronavirus OC43, anastrozole, NLRP3 inflammasome, NF-κB, pyroptosis

## Abstract

The common human coronavirus (HCoV) exhibits mild disease with upper respiratory infection and common cold symptoms. HCoV-OC43, one of the HCoVs, can be used to screen drug candidates against SARS-CoV-2. We determined the antiviral effects of FDA/EMA-approved drug anastrozole (AZ) on two human coronaviruses, HCoV-OC43 and HCoV-229E, using MRC-5 cells in vitro. The AZ exhibited antiviral effects against HCoV-OC43 and HCoV-229E infection. Subsequent studies focused on HCoV-OC43, which is related to the SARS-CoV-2 family. AZ exhibited anti-viral effects and reduced the secretion of inflammatory cytokines, TNF-α, IL-6, and IL-1β. It also inhibited NF-κB translocation to effectively suppress the inflammatory response. AZ reduced intracellular calcium and reactive oxygen species (ROS) levels, including mitochondrial ROS and Ca^2+^, induced by the virus. AZ inhibited the expression of NLRP3 inflammasome components and cleaved IL-1β, suggesting that it blocks NLRP3 inflammasome activation in HCoV-OC43-infected cells. Moreover, AZ enhanced cell viability and reduced the expression of cleaved gasdermin D (GSDMD), a marker of pyroptosis. Overall, we demonstrated that AZ exhibits antiviral activity against HCoV-OC43 and HCoV-229E. We specifically focused on its efficacy against HCoV-OC43 and showed its potential to reduce inflammation, inhibit NLRP3 inflammasome activation, mitigate mitochondrial dysfunction, and suppress pyroptosis in infected cells.

## 1. Introduction

Human coronaviruses (HCoVs), which were first identified in the mid-1960s, are positive-stranded RNA viruses that, under an electron microscope, exhibit crown-like spikes on their surface [1,2]. Four common types, including HCoV-OC43, -229E, -NL63, and -HKU1, which usually cause mild-to-moderate upper respiratory tract infections, and three highly pathogenic types of viruses, including severe acute respiratory syndrome (SARS)-CoV, Middle-East respiratory syndrome (MERS)-CoV, and SARS-CoV-2, which cause severe respiratory diseases, have been recognized. Based on a genomic analysis, CoVs may be subdivided into four genera: alpha, beta, delta, and gamma. HCoVs belong to the alpha coronavirus (-229E, -NL63) and beta coronavirus (-OC43, -HKU1, SARS-CoV, MERS-CoV, and SARS-CoV-2) groups. Common HCoVs are considered responsible for up to a third of common cold cases [3,4,5,6]. In particular, one of the endemic strains of low-risk coronaviruses, HCoV-OC43, is a beta coronavirus, similar to SARS-CoV-2, which is the pathogen responsible for the ongoing COVID-19 pandemic. Despite its limitations in causing only mild disease and upper respiratory infection, HCoV-OC43 has potential as a surrogate for screening drug candidates for SARS-CoV-2 treatment, which can help avoid the added costs and biosafety concerns of biosafety level (BSL)-3 protocols required for SARS-CoV-2 research [5,7].

Viral infections affect the production and release of reactive oxygen species within the mitochondria (mtROS), resulting in mitochondrial dysfunction [7,8,9,10,11,12,13,14,15]. Although ROS are chemically reactive molecules that play important roles in cell signaling and homeostasis, excessive ROS results in oxidative stress and promotes an inflammatory response and cell death [16,17]. In particular, ROS activates nuclear factor kappa B (NF-κB), which translocates to the nucleus to regulate the expression of various genes, including proinflammatory factors in response to oxidative stress conditions. Virus-induced mtROS can cause an excessive immune response through the formation of the nod-like receptor protein 3 (NLRP3) inflammasome, which is an intracellular sensor comprising NLRP3, apoptosis-associated speck-like protein containing a CARD (ASC), and precursor caspase-1 (CASP1), which can detect a wide range of stimuli, ultimately resulting in cellular damage [18,19]. Caspase-1 activation, following further processing by precursor caspase-1, cleaves prointerleukin-1β (pro-IL-1β), pro-IL-18, and gasdermin D (GSDMD), consequently forming transmembrane pores with the N-terminus of the GSDMD (GSDMD-N) and facilitating the release of proinflammatory cytokines, such as IL-1β and IL-18, and the dysregulation of intracellular and extracellular ion gradients, exacerbating inflammation and ultimately triggering pyroptosis following viral infection [20,21]. Thus, compounds targeting ROS and the ROS-mediated inflammatory response represent potential antiviral agents.

Anastrozole (AZ), a nonsteroidal drug approved by the U.S. Food and Drug Administration (FDA) and the European Medicines Agency (EMA), is prescribed for the treatment of breast cancer. Although human breast epithelial cells can proliferate in response to stimulation by female hormones, such as estrogen, overexposure to estrogen can increase the likelihood of developing cancer [22,23,24]. Estrogen is primarily produced in postmenopausal women by the enzyme aromatase, which is abundant in fat cells [25,26]. AZ inhibits the production of estrogen by competitively binding to the aromatase enzyme, which converts androgens to estrogens, to reduce tumor proliferation and growth in hormone receptor-positive breast cancer [27,28].

Previous research suggested the potential of AZ on anti-COVID-19 activity through estrogen regulation and its effect on TMPRSS2 signaling [29,30]. However, these suggestions remain speculative, requiring further scientific validation. Due to limitations in using BSL-3 facilities for SARS-CoV-2 research, we investigated AZ’s antiviral effects against human coronaviruses similar to SARS-CoV-2, identifying distinct mechanisms from estrogens. Therefore, in this study, we assessed the antiviral potential of AZ and investigated its antiviral mechanisms against HCoV (HCoV-OC43 and -229E) infection, primarily focusing on the regulation of NLRP3 inflammasome, mitochondrial stress, and pyroptosis inhibition during HCoV infection.

## 2. Materials and Methods

### 2.1. Reagents

AZ was purchased from Tocris Bioscience (Bristol, UK). Minimum Essential Medium (MEM), fetal bovine serum (FBS), and penicillin/streptomycin (P/S) were purchased from Hyclone (Pittsburgh, PA, USA). The enhanced chemiluminescence (ECL), nuclear/cytoplasm isolation kit, mitochondrial superoxide indicators (MitoSOX), 2,7-dichlorofluorescein diacetate (DCF-DA), Fluo-4 acetoxymethyl ester (Fluo-4), rhod-2 acetoxymethyl ester (Rhod-2), 3,3′-dihexyloxacarbocyanine iodide [DioC6(3)], HCoV-OC43 nucleoprotein (N protein) polyclonal antibody, and HCoV-229E nucleoprotein polyclonal antibody were obtained from Thermo Fisher Scientific (Waltham, MA, USA). The PRO-PREP protein extraction solution was purchased from Intron Biotechnology (Seoul, Republic of Korea). The 3-(4,5-dimethylthiazol-2-yl)-2,5-diphenyltetrazolium bromide (MTT), molnupiravir (EIDD-2801, EIDD), and DMSO were purchased from Sigma-Aldrich (St. Louis, MO, USA). IL-6, TNF-α, and IL-1β ELISA kits were purchased from BD Biosciences (San Diego, CA, USA). Cleaved caspase-1 (1:1000; Cat. 4199S, Rabbit IgG), gasdermin D (GSDMD; 1:1000; Cat. 39754S, Rabbit IgG), cleaved gasdermin D (cleaved GSDMD; 1:1000; Cat. 36425S, Rabbit IgG), cleaved IL-1β (1:1000; Cat. 63124S, Rabbit IgG), nod-like receptor protein 3 (NLRP3; 1:1000; Cat. 15101S, Rabbit IgG), apoptosis-associated speck-like protein containing a CARD (ASC; 1:1000; Cat. 13833S, Rabbit IgG), phosphorylated nuclear factor kappa B (p-NF-κB; 1:500; Cat. 3033S, Alexa-488-rabbit IgG), and β-actin (1:1000; Cat. 12262S, Mouse IgG) antibodies were purchased from Cell Signaling Technology (Danvers, MA, USA).

### 2.2. Cells and Viruses

MRC-5 cells were grown in MEM supplemented with 10% FBS and 1% P/S (100 U/mL) at 37 °C in a humidified incubator with an atmosphere containing 95% air and 5% CO_2_. The HCoV-OC43 and HCoV-229E strains were used as previously described [31].

### 2.3. Antiviral Effect

MRC-5 cells were cultured in 24-well plates at a density of 1 × 10^5^ cells/well for 18 h. For the co-infection assay, various concentrations of AZ and HCoV-OC43 (MOI = 0.1) or HCoV-229E (MOI = 2), were mixed and incubated at 4 °C for 3 h, respectively. After incubation, the cells were exposed to the mixture at 4 °C for 3 h, washed, and added to complete media. For the post-treatment assay, the cells were exposed to HCoV-OC43 and HCoV-229E for 3 h, washed, and treated with various concentrations of AZ for 24 h. After incubation, N protein staining was performed according to Section 2.4.

### 2.4. Immunofluorescence Staining

The cells were fixed with 4% paraformaldehyde in phosphate-buffered saline (PBS) for 10 min at 25 °C, washed three times with PBS, incubated with a 0.5× Ez-Blocking solution for 1 h, followed by incubation with N protein and phosphorylated NF-κB antibodies overnight at 4 °C. The cells were washed with PBS and incubated with a secondary antibody for 2 h on a rocker in a dark room. Finally, the cells were washed with PBS and stained with Hoechst 33342 for 15 min. The cultured wells were mounted on slides using a mounting medium and visualized using a fluorescence microscope (Nikon Corporation, Tokyo, Japan).

### 2.5. Calculation of Selectivity Index

The selectivity index (SI) indicates the relative effectiveness of a drug in inhibiting viral replication compared to inducing cell death. SI calculations were made as the ratio of the 50% cytotoxic concentration (CC_50_) and the 50% inhibitory concentration (IC_50_). The formula is: SI = IC_50_/CC_50_ This recommends an acceptance criterion of SI ≥ 10 for selective bioactive samples.

### 2.6. Western Blot Analysis

Western blot analysis was performed as described using our standard operating protocol [30]. Briefly, equivalent amounts of protein were separated using sodium dodecyl sulfate-polyacrylamide gel electrophoresis (SDS-PAGE) and transferred to polyvinylidene fluoride (PVDF) membranes. The membranes were incubated in a blocking solution followed by incubation with the antibody (primary antibody to secondary antibody). Protein bands were detected using a ChemiDoc imaging system (UVITEC, Cleaver Scientific Ltd., Warwickshire, UK) with an enhanced chemiluminescence reagent (BioRad, Richmond, CA, USA).

### 2.7. Fluorescence Analysis

Intracellular Ca^2+^ and ROS generation were analyzed by flow cytometry using DCF-DA and Fluo-4. Mitochondria1 ROS and mitochondrial membrane potential (MMP, Δψm) were determined using MitoSOX and DioC6(3). Staining was done according to the manufacturer’s instructions, and fluorescence was analyzed using CytoFLEX (Beckman Coulter Inc., Pasadena, CA, USA).

### 2.8. Inflammatory Cytokines

Human TNF-α, IL-6, and IL-1β levels in the supernatants were measured using ELISA kits according to the manufacturer’s instructions.

### 2.9. Statistical Analysis

All the experiment data are expressed as the mean ± SEM. The significance of the differences in the mean values between the treated and control groups was determined using a one-way analysis of variance. Tukey’s post hoc test was used for multigroup comparisons. The analyses were performed using GraphPad PRISM Version 8.01 (GraphPad, San Diego, CA, USA) software, and *p*-values < 0.05 were considered statistically significant.

## 3. Results

### 3.1. Antiviral Effect of AZ on HCoV-OC43 and HCoV-229E in MRC-5 Cells

**Figure 1:**
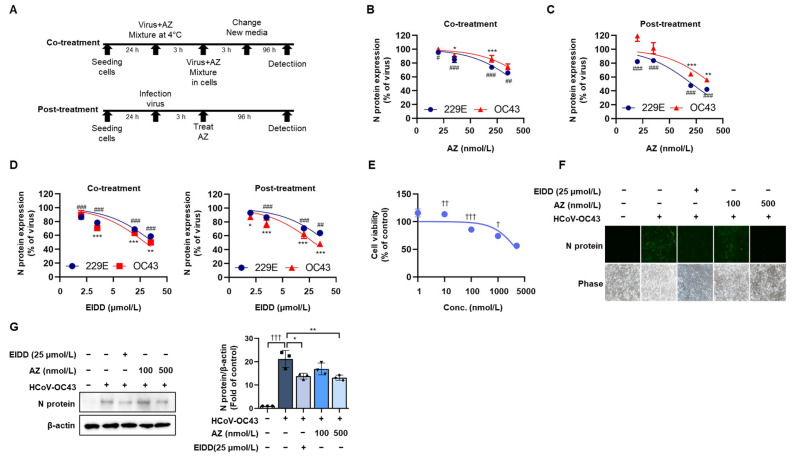
Antiviral effect of AZ against HCoV-OC43 and HCoV-229E. (**A**) Scheme of the treatment conditions for co- and/or post-treatment HCoV assays. (**B**) In the co-treatment assay, a mixture of HCoV-OC43 or -229E and AZ was incubated at 4 °C for 3 h and then added to MRC-5 cells. (**C**) For the post-treatment assay, cells were infected with HCoV-OC43 or -229E and then treated with AZ. (**D**) Antiviral effect of EIDD under co- and post-treatment assay conditions. EIDD was used as a positive control. (**E**) MRC-5 cells were treated with AZ at 37 °C for 96 h and subject to an MTT assay. For the post-treatment assay, the cells were infected with HCoV-OC43 and then treated with AZ or EIDD (25 μmol/L). After the reaction, the detection of the viral N protein was conducted by (**F**) immunofluorescence assay and (**G**) Western blot analysis. The image shows the N protein (Green). The size of the scale bar is 100 µm. The band was quantitated using Image J software 1.5.4. Three independent experiments were performed. Bar graph (mean ± standard error of mean) statistical data were determined using a one-way analysis of variance with Tukey’s post hoc test. *** *p* < 0.001; ** *p* < 0.01; and * *p* < 0.05 compared with the HCoV-OC43 infection group. ### *p* < 0.001; ## *p* < 0.01; and # *p* < 0.05, compared with the HCoV-229E infection groups. ††† *p* < 0.001; †† *p* < 0.01; and † *p* < 0.05, compared with the untreated group.

We examined the anti-HCoV-OC43 and anti-HCoV-229E activity of AZ under co-treatment and post-treatment conditions (Figure 1A). In the co-treatment assay, AZ and HCoV (HCoV-OC43 or HCoV-229E) were mixed and incubated at 4 °C for 3 h and then used to infect MRC-5 cells. After the reaction, the expression of viral N protein in both HCoV-treated cells was slightly decreased by the AZ (Figure 1B). For the post-treatment assay, the cells were infected with HCoV-OC43 and HCoV-229E for 3 h, followed by treatment with AZ. The expression of viral N protein in HCoV-OC43- and HCoV-229E-treated cells was suppressed by the AZ (Figure 1C). In addition, we used EIDD as a positive control. EIDD decreased the expression of N protein in both co- and post-treatment assays (Figure 1D). Cell viability was also confirmed by treatment with AZ after calculating the 50% cytotoxic concentration (CC_50_) of the AZ, which was 5.1 μmol/L (Figure 1E).

The selectivity index (SI) was calculated to confirm the antiviral activity of AZ against HCoV-OC43 and HCoV-229E in the in vitro assay. SI refers to the relative effectiveness of a drug at inhibiting viral replication compared with the induction of cell death. Compounds with an SI value ≥ 10 are generally considered to be active in vitro. Based on this formula, the SI values of AZ for HCoV-OC43 and HCoV-229E in the co-treatment assay were 3.5 and 6.3, respectively, whereas the SI values of AZ of HCoV-OC43 and HCoV-229E in the post-treatment assay were 8.0 and 19.1, respectively. Moreover, the SI values of EIDD for HCoV-OC43 and HCoV-229E in the co-treatment assay were 4.8 and 3.3, and 4.8 and 2.6 in the post-treatment assay, respectively (Table 1). The results indicate that AZ exhibits a higher antiviral activity for HCoV-229E compared with HCoV-OC43; however, we focused on HCoV-OC43, which belongs to the same beta coronavirus family as SARS-CoV-2 [4]. We further examined the antiviral effects of AZ against HCoV-OC43 under post-treatment conditions. The expression of N protein was measured by immunofluorescence and Western blot analysis (Figure 1F,G). AZ reduced the expression of the N protein, indicating that AZ inhibits infection by HCoV-OC43 in MRC-5 cells.

### 3.2. AZ Reduces the Inflammatory Response in HCoV-OC43-Infected MRC-5 Cells

**Figure 2:**
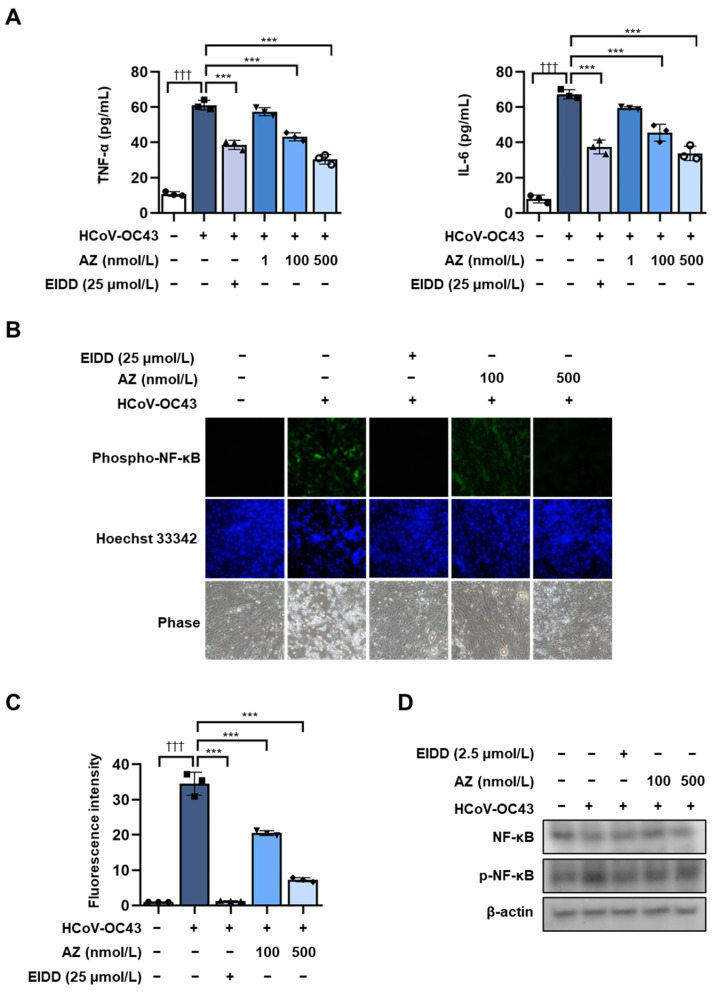
Anti-inflammatory effect of AZ on HCoV-OC43-infected MRC-5 cells. (**A**) The secretion of inflammatory cytokines, including TNF-α and IL-6, was determined with ELISA kits. (**B**) Immunofluorescence was performed to detect NF-κB translocation to the nucleus. The image shows p-NF-κB (Green) and Hoechst 33342 (Blue) staining. (**C**) Fluorescence intensity was quantitated using ImageJ software. The scale bar is 100 µm. (**D**) NF-κB detection using Western blot. Bar graph (mean ± standard error of mean, n = 3) statistical data were determined using a one-way analysis of variance with Tukey’s post hoc test. ††† *p* < 0.001 compared with the untreated group. *** *p* < 0.001 compared with the HCoV-OC43 infection group.

HCoV-OC43 infection directly or indirectly up-regulates inflammatory cytokines, including TNF-α, IL-6, and IL-1β, thus exacerbating inflammation through an inflammatory cascade [32]. We confirmed the secretion of these inflammatory cytokines using ELISA kits at the cellular level in HCoV-OC43-infected MRC-5 cells. As shown in Figure 2A, AZ reduced the increase in inflammatory cytokines following HCoV-OC43 infection. NF-κB is well known to promote the inflammatory response. Therefore, we determined the effect of AZ on the translocation of NF-κB to the nucleus. The results indicated that HCoV-OC43 infection increased the expression of phospho-NF-κB in the nucleus, whereas AZ decreased translocation (Figure 2B–D). Therefore, these data indicate that AZ inhibits the inflammatory response in HCoV-OC43-infected MRC-5 cells by suppressing NF-κB activity.

### 3.3. AZ Suppresses the Expression of the NLRP3 Inflammasome in HCoV-OC43-Infected Cells

**Figure 3:**
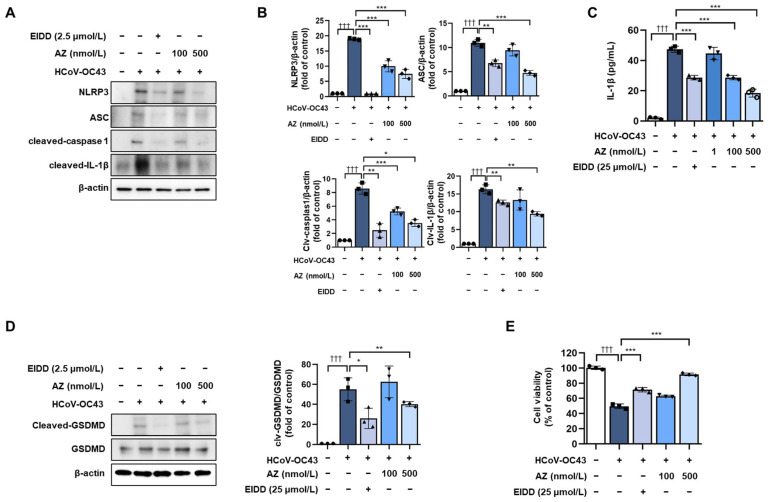
AZ decreases the expression of the NLRP3 inflammasome complex in HCoV-OC43-infected MRC-5 cells. (**A**) Expression of the inflammasome components, NLRP3, ACS, and cleaved caspase-1, as well as cleaved IL-1β, were measured by Western blot analysis. (**B**) Quantitative analysis of the protein bands was done using ImageJ software. (**C**) The secretion of inflammatory cytokines such as IL-1β was determined with ELISA kits. (**D**) GSDMD expression was measured by Western blot analysis. (**E**) Cell viability was measured using the MTT assay. Bar graph (mean ± standard error of mean, n = 2) statistical data were determined using a one-way analysis of variance with Tukey’s post hoc test. ††† *p* < 0.001 compared with the untreated group. *** *p* < 0.001; ** *p* < 0.01; and * *p* < 0.05 compared with the HCoV-OC43 infection group.

The NLRP3 inflammasome is activated following viral infection [18]. The components of the NLRP3 inflammasome include NLRP3, ASC, and caspase-1. These complexes assemble in response to diverse stimuli, resulting in the activation of caspase-1. Activated caspase-1 converts pro-IL-1β to IL-1β to induce a proinflammatory response [33]. Therefore, we determined the effect of AZ on preventing the activation of the NLRP3 inflammasome in HCoV-OC43-infected MRC-5 cells. As shown in Figure 3A–C, AZ inhibited the increased expression of the NLRP3 inflammasome components, NLRP3, ASC, and cleaved caspase-1, and reduced both the secretion of IL-1β and the expression of cleaved IL-1β following HCoV-OC43 infection. The results indicate that AZ decreased the expression of the NLRP3 inflammasome in HCoV-OC43-infected MRC-5 cells. Pyroptosis is activated downstream of inflammasome activation. The activation of caspase-1 can cleave the pore-forming protein gasdermin D (GSDMD), freeing its N-terminus, which targets the cell membrane to assemble pore structures and induce pyroptosis, a form of inflammatory cell death. In addition, the expression of the cleaved GSDMD, a marker of pyroptosis, was measured via Western blot analysis. The results indicated that the expression of cleaved GSDMD increased by HCoV-OC43 infection decreased after the AZ treatment (Figure 3D) [34,35,36]. We determined the effect of AZ on reducing cell viability following HCoV-OC43 infection. AZ treatment increased the cell survival rate, which was reduced by viral infection (Figure 3E). The results suggest that AZ inhibits HCoV-OC43-induced pyroptosis to maintain cell viability.

### 3.4. AZ Reduces HCoV-OC43-Induced Mitochondria Stress in MRC-5 Cells

**Figure 4:**
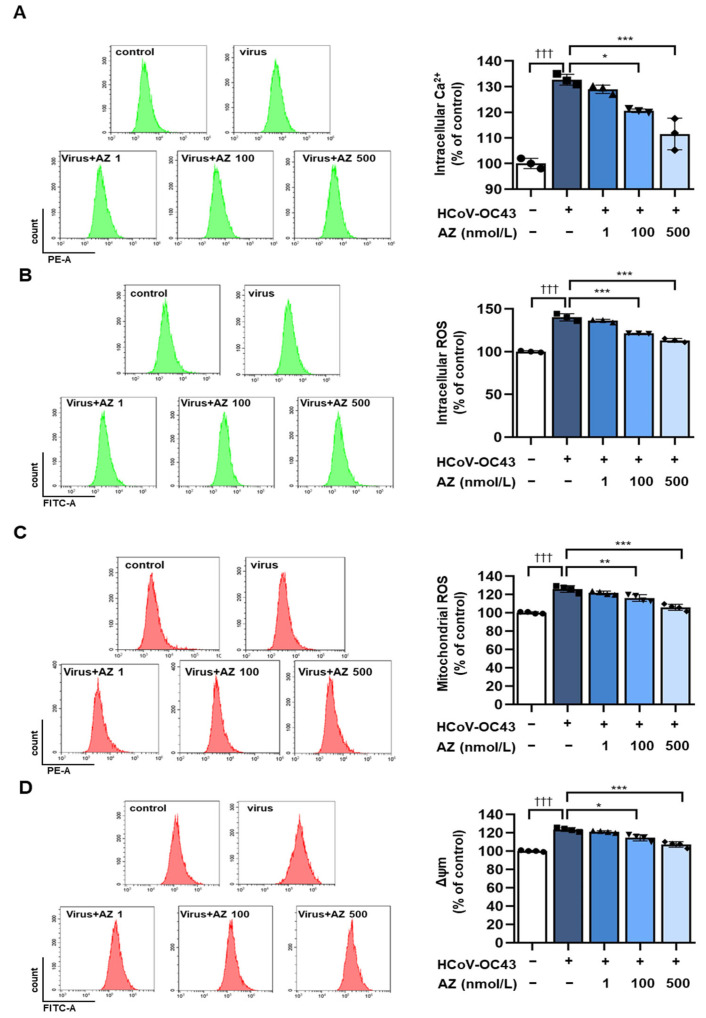
AZ decreases HCoV-OC43-induced mitochondria stress in MRC-5 cells. Cells were infected with HCoV-OC43 for 3 h, treated with AZ for 72 h, and stained with a fluorescent dye. (**A**) Intracellular Ca^2+^ was measured by Flou-4. (**B**) Intracellular H_2_O_2_ was determined using DCF-DA. (**C**) Mitochondrial ROS (mtROS) was analyzed using MitoSOX as a selective mtROS detection dye. (**D**) Mitochondrial membrane potential (MMP, Δψm) was measured using DioC6(3). (**A**–**D**) show histograms for flow cytometry and graphs quantifying fluorescence. Bar graph (mean ± standard error of mean) statistical data were determined using a one-way analysis of variance with Tukey’s post hoc test. ††† *p* < 0.001 compared with the untreated group. *** *p* < 0.001; ** *p* < 0.01; and * *p* < 0.05 compared with the HCoV-OC43 infection group.

HCoV-OC43 infection induces oxidative stress by generating ROS and Ca^2+^, resulting in mitochondrial dysfunction. Excessive oxidative stress can increase the cellular inflammation response, including proinflammatory pathways and NF-κB activity. In addition, ROS and mtROS drive viral replication and apoptosis [35]. Therefore, we measured changes in intracellular calcium and ROS following HCoV-OC43 infection. The results indicated that viral infection increased intracellular calcium and ROS, which reduced in a dose-dependent manner after the AZ treatment (Figure 4A,B). The inhibitory effect of AZ on HCoV-OC43-induced mitochondrial dysfunction was determined. AZ treatment reduced mtROS and Ca^2+^, which were increased by the HCoV-OC43 infection (Figure 4C,D).

## 4. Discussion

Some FDA/EMA-approved drugs, including those initially developed for a specific medical condition, show potential for treating diseases or conditions beyond their original intended use [37,38]. Herein, we examined the antiviral effect of AZ, which is an aromatase inhibitor used to treat hormone receptor-positive breast cancer in postmenopausal women [39,40]. Previous studies have predicted that AZ may exhibit anti-COVID-19 activity by regulating estrogen production and following p65-transmembrane serine protease 2 (TMPRSS2) signaling. While there have been some studies and hypotheses suggesting its potential to affect certain mechanisms related to SARS-CoV-2, particularly in the context of estrogen regulation and its impact on TMPRSS2 signaling, these studies remain largely speculative and in need of further scientific investigation for validation [29,30]. Therefore, it is imperative to identify the scientific mechanism for the anti-SARS-CoV-2 effect of AZ. Unfortunately, there are limitations in using BSL-3 facilities for SARS-CoV-2 research, so we investigated the antiviral effects of AZ against human coronaviruses similar to SARS-CoV-2 and the antiviral mechanisms that distinguish it from estrogens. In addition, antiviral research against coronaviruses that infect humans, such as HCoVs and SARS-CoV-2, has been continuously required due to the diverse viral mutations that occur in RNA viruses. Until recently, there were no reports regarding the antiviral effect of AZ against HCoV. Therefore, to the best of our knowledge, our study is the first to confirm the effect of AZ on HcoV, as well as the underlying mechanism for its antiviral activity.

**Figure 5:**
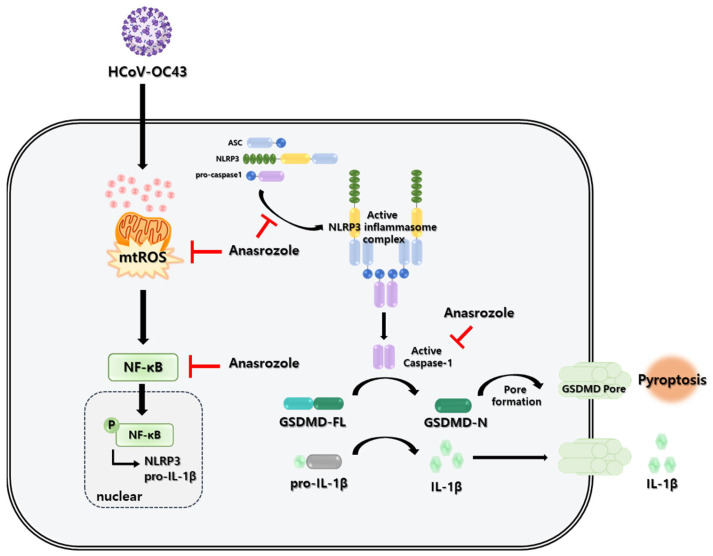
Effect of AZ on HCoV-OC43 infection. AZ inhibits HCoV-OC43 infection by pyroptosis through the ROS-NLRP3 inflammasome pathway.

We demonstrated that AZ inhibits HCoV-OC43 infection and ROS-mediated NLRP3 inflammasome, which is increased in HCoV-OC43-infected cells. The results indicate that AZ decreased the expression of HCoV-OC43 viral proteins, such as the N protein. In addition, AZ suppressed intracellular ROS and calcium levels, which were increased by the viral infection. Excess ROS induced by HCoV-OC43 infection results in the accumulation of mitochondrial dysfunction; however, this effect is reduced by AZ treatment. ROS is closely associated with the inflammatory response [41,42]. Because we previously confirmed that AZ reduces ROS levels induced by the viral infection, we predicted that AZ would also be effective against HCoV-OC43-induced inflammation. As expected, AZ reduced inflammatory cytokine expression, including TNF-α and IL-1β, and the translocation of NF-κB to the nucleus, which are all increased by viral infection. Furthermore, there was an inhibitory effect of AZ on the expression of components of the NLRP3 inflammasome complex (NLRP3, caspase-1, and ASC) in HCoV-OC43-infected MRC-5 cells (Figure 5).

In addition, HCoV, as a single-stranded RNA (ssRNA) virus, is recognized by Toll-like receptors TLR-7/-8 and RIG-I-like receptors (RLRs) [43]. Recognition of HCoV by TLRs and RLRs triggers intracellular signaling cascades, leading to the activation of the transcription factor NF-κB [35]. NF-κB activation results in the transcription of genes involved in the production of pro-inflammatory cytokines and the induction of type 1 interferons. This effectively captures the key aspects of how the immune system responds to HCoV infection through TLRs and RLRs, highlighting the activation of NF-κB, pro-inflammatory cytokine production, and the induction of type I interferons as crucial components of the antiviral defense mechanism. We describe an experimental observation suggesting that AZ treatment reduces the increased expression of RIG-1 and IFN-β secretion caused by HCoV-OC43 infection (Supplementary Figures S1 and S2). Therefore, we propose that AZ may regulate the NLRP3 inflammasome by inhibiting RIG-1.

Moreover, the upregulation of RIG-1 induces the activation of the NLRP3 inflammasome. The activation of the NLRP3 inflammasome results in pyroptosis, a robust inflammatory cell death process, during HCoV-OC43 infection [44,45,46,47]. Pyroptosis is executed by gasdermin family members, which are activated through inflammasome activation-mediated caspase-1 cleavage of gasdermin D (GSDMD). We demonstrated that viral infection induces cleavage of GSDMD by increasing cleaved caspase-1 levels, and AZ has been shown to inhibit this process. Collectively, our findings demonstrate that pyroptosis is triggered by human coronavirus infection, and NLRP3 inflammasome dysfunction may result in negative consequences for the host.

In this study, we reported that the heightened ROS induced by viral infection activates the NLRP3 inflammasome, leading to GSDMD activation. However, a previous study indicated that GSDMD causes rapid damage to both the inner and outer mitochondrial membranes, resulting in the generation of ROS, loss of transmembrane potential, and impairment of oxidative phosphorylation [48]. The interplay between these processes could contribute to the overall cellular response to viral infection, potentially linking inflammation, cell death, and mitochondrial dysfunction. Further investigation would be necessary to understand the specific mechanisms and consequences of these interactions in the context of viral infections.

In addition, inflammatory cell death includes necroptosis and apoptosis, in addition to pyroptosis. This concept of PANoptosis has been established based on numerous reports demonstrating crosstalk between inflammasome-mediated pyroptosis, apoptosis, and necroptosis [49,50]. PANoptosis is a unique inflammatory programmed cell death pathway regulated by the PANoptosome, which provides a molecular scaffold that enables the interaction and activation of the machinery required for inflammasome/pyroptosis (such as NLRP3, ASC, and caspase-1), apoptosis (caspase-8), and necroptosis (RIPK3/RIPK1) [51,52]. Additional studies are warranted to determine the effect of HCoV-OC43 infection on other mechanisms of inflammatory cell death and the impact of AZ.

Our results suggest that new targets and treatment approaches can be found through studies using a drug with multiple indications for various diseases, thus strengthening the existing drug paradigm. In addition, preemptively assessing the antiviral efficacy with various currently available drugs is crucial to prepare for both current and newly emerging coronavirus variants, and enhancing research efficiency can be achieved by using HCoVs similar to SARS-CoV-2 as surrogates for SARS-CoV-2, particularly due to facility limitations. Beyond the previously suggested estrogen-associated anti-COVID-19 effect of AZ in breast cancer patients, additional investigation into its various antiviral mechanisms against HCoVs may provide crucial information for expanding its therapeutic uses. Taken together, we demonstrated that AZ is effective on HCoV-OC43 infection and inhibits pyroptosis through suppression of the ROS-mediated NLRP3 inflammasome pathway, thereby promoting cell viability. Based on our results, we suggest that AZ has potential as a new and effective therapeutic agent with antiviral effects against HCoV-OC43.

## 5. Conclusions

We demonstrated the antiviral effect of FDA/EMA-approved drug AZ against HCoVs by evaluating the inhibition of viral infection by its treatment and elucidating its antiviral mechanisms. Our findings showed that AZ is effective against both HCoV-OC43 and HCoV-229E infection. Specifically, AZ inhibits pyroptosis by attenuating the ROS-mediated NLRP3 inflammasome pathway, consequently mitigating a decrease in cell viability, which is caused by HCoV-OC43 infection. This suggests that AZ appears to be a potentially beneficial medication with novel therapeutic and antiviral efficacy against HCoV-OC43 because of its ability to modulate ROS and NLRP3 inflammasomes.

## Figures and Tables

**Table 1:** Selectivity index of AZ and EIDD against HCoVs.

Viruses	Compounds	Co-Treatment		Post-Treatment	
		IC_50_ (nM)	SI *	IC_50_ (nM)	SI
HCoV-OC43	AZ	1451 ± 4.5	3.5	632.2 ± 14.3	8.0
	EIDD **	38,885 ± 10.7	4.8	38,943 ± 8.3	4.8
HCoV-229E	AZ	803.2 ± 5.9	6.3	265.1 ± 6.7	19.1
	EIDD	55,955 ± 9.1	3.3	72,126 ± 5.5	2.6

* SI: Selectivity Index. ** EIDD: EIDD-2801.

## Data Availability

All the data is available within the article.

## Supplementary Materials

The following supporting information can be downloaded at: https://www.mdpi.com/article/10.3390/antiox13010116/s1.
