# Supplementary material for: Anastrozole Protects against Human Coronavirus Infection by Ameliorating the Reactive Oxygen Species–Mediated Inflammatory Response
Source: Antioxidants (Basel). 2024 Jan 17;13(1):116. doi: 10.3390/antiox13010116 (PMC10813058; doi:10.3390/antiox13010116)
Supplement: Supplementary file 1 [file antioxidants-13-00116-s001.zip › antioxidants-2763636-supplementary.pdf]

Supplementary figures

Anastrozole Protects against Human Coronavirus Infection by Ameliorating the Reactive Oxygen Species–Mediated Inflammatory Response

Eun-Bin Kwon, Buyun Kim, Young Soo Kim * and Jang-Gi Choi *

Korean Medicine (KM) Application Center, Korea Institute of Oriental Medicine (KIOM), Dong-gu, Daegu 41062, Republic of Korea; wrld2931@kiom.re.kr (E.-B.K.); bykim@kiom.re.kr (B.K.)
* Correspondence: yskim527@kiom.re.kr (Y.S.K.); jang-gichoi@kiom.re.kr (J.-G.C.)

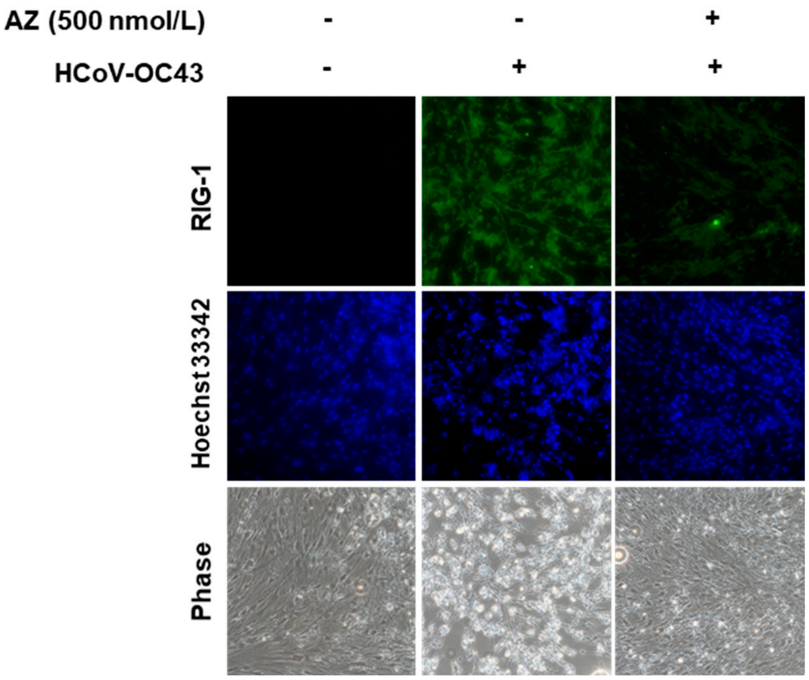

Figure S1. AZ reduced HCoV-OC43-induced RIG-1 expression. Expression of RIG-1 was detected via immunofluorescence.

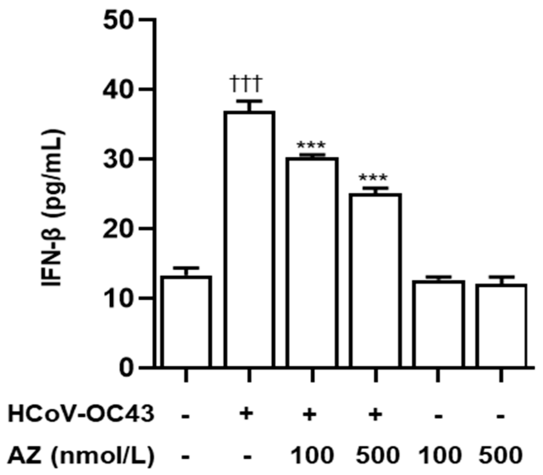

Figure S2. Effect of AZ on the secretion of HCoV-OC43-induced IFN-β. MRC-5 cells were infected with HCoV-OC43 for 3 h and were treated with AZ for 72 h. Then, and the supernatant was harvested. The secretion of IFN-β was determined with ELISA kits. †††P <0.001 compared with the untreated group. ***P < 0.001 compared with the HCoV-OC43 infection group.
